# Identification of potential biomarkers related to mannose metabolism in keloids: analysis of integrated bulk RNA-seq and scRNA-seq

**DOI:** 10.3389/fimmu.2026.1711588

**Published:** 2026-06-10

**Authors:** Jiaoquan Chen, Xiaoyu Xiong, Bihua Liang, Yeqing Gong, Shaoyin Ma, Xin Zhou, Huilan Zhu, Ling Lin, Rihua Lin

**Affiliations:** Department of Dermatology, Guangzhou Dermatology Hospital, Guangzhou, China

**Keywords:** biomarkers, fibroblasts, keloid, keratinocytes, mannose metabolism

## Abstract

**Background:**

Keloid (KD) is a benign cutaneous fibrotic disorder characterized by excessive proliferation of dermal fibroblasts. Mannose plays a key role in cellular metabolism, yet its specific mechanism associated with KD remains unclear. Therefore, identifying mannose metabolism-related potential biomarkers and their regulatory mechanisms in KD is crucial.

**Methods:**

Differentially expressed genes (DEGs) were identified between KD and control samples, and their intersection with mannose metabolism-related genes (MMRGs) was obtained to determine candidate genes. Feature genes underwent machine learning-based screening to identify characteristic genes, while potential biomarkers were determined through integrated analysis of gene expression profiles and Receiver Operating Characteristic (ROC) curve assessment. Subsequently, reverse transcription-quantitative polymerase chain reaction (RT-qPCR) methodology was employed to validate the expression patterns of these identified potential biomarkers. Subsequently, nomogram construction and enrichment analysis were conducted. Finally, key cells were identified through single-cell analysis, followed by performing cell communication, pseudotime, and transcription factor regulation analyses.

**Results:**

A total of 1,372 DEGs were identified, from which two mannose metabolism-related potential biomarkers (*MANBA* and *TMTC2*) in KD were further screened out. RT-qPCR results confirmed that these two potential biomarkers were significantly upregulated in the KD group. The nomogram prediction model developed utilizing these potential biomarkers demonstrated favourable clinical prognostic capabilities. Pathway enrichment analysis revealed that both identified potential biomarkers exhibited significant co-enrichment patterns across various biological pathways, including cellular cycle regulation processes. Single-cell analysis results indicated that fibroblasts and keratinocytes played key roles in the progression of KD, and dynamic expression changes of MANBA and TMTC2 were observed during the differentiation of these two cell types. In fibroblast subtypes, the expression levels of transcription factors such as *JUNB* (+) were relatively high, while in keratinocyte subtypes, the expression level of *FOSL1* (+) was relatively high.

**Conclusion:**

This study successfully identified two potential biomarkers (*MANBA* and *TMTC2*) and two key cell types (fibroblasts and keratinocytes), providing new insights into potential therapeutic strategies for KD.

## 1 Introduction

Keloid (KD) is a recalcitrant fibroproliferative disorder characterized by excessive and invasive deposition of collagen and extracellular matrix (ECM) beyond the original wound margins. These raised, firm, and often erythematous lesions, which frequently exhibit crab-claw-like projections, commonly occur on the chest, shoulders, and earlobes in humans (1–3). They cause substantial physical discomfort via pruritus and pain in KD patients, alongside significant psychosocial distress (4). The pathogenesis of KD is complex and multifactorial, involving a strong genetic predisposition, dysregulated proliferation and apoptosis of fibroblasts, aberrant crosstalk between keratinocytes, fibroblasts, and mast cells, and a sustained inflammatory response that correlates with disease severity (5–7). Current mainstay therapies, including surgical excision, radiotherapy, intralesional corticosteroids, and topical agents, are associated with high recurrence rates and significant adverse effects (8, 9), which highlights a pressing clinical need for novel, targeted treatment strategies. This necessitates a deeper understanding of the molecular mechanisms driving pathological KD formation and the identification of reliable biomarkers for diagnosis, therapeutic targeting, and intervention.

Mannose, a hexose sugar that is metabolized primarily to mannose-6-phosphate (M6P), functions not merely as an energy source; it is a potential key regulator of inflammation and ECM synthesis (10–13). Studies have shown that KD fibroblasts exhibit altered mannose consumption patterns (14). Exogenous mannose supplementation can dose-dependently suppress the expression of collagen types I and III in these cells, an effect potentially mediated by competition with glucose metabolism, leading to reduced ATP availability and a subsequent inhibition of fibroblast proliferation and collagen production (15). The key metabolite M6P has been suggested to exert anti-fibrotic effects, possibly by inhibiting the expression of the master pro-fibrotic cytokine TGF-β1 in KD fibroblasts (16, 17). Furthermore, mannose may attenuate the chronic inflammatory milieu in KD by suppressing the NF-κB signalling pathway, thereby reducing the release of pro-inflammatory mediators such as IL-6 and TNF-α (18). The previously observed upregulation of the insulin-like growth factor-II/mannose-6-phosphate receptor (IGF-II/M6PR) in KD fibroblasts further underscores a potential link between mannose metabolism and disease pathogenesis (19). Despite these compelling associations, the precise molecular mechanisms linking mannose metabolism to KD development at the cellular and molecular levels remain largely unexplored and require systematic elucidation.

The recent advent of single-cell RNA sequencing (scRNA-seq) provides a powerful tool to dissect the complexity of unclear links between mannose metabolism and KD pathogenesis at an unprecedented resolution (20). Unlike traditional bulk RNA-seq, which averages gene expression across an entire tissue, scRNA-seq enables the precise characterization of individual cells within the heterogeneous pathological KD tissue (21). This technology is instrumental in identifying distinct cellular subpopulations, such as pro-fibrotic myofibroblast subsets, delineating the functional states of immune cells, and mapping intricate cell-cell communication networks that drive KD-related fibrosis (22, 23). In recent years, multiple studies have utilized scRNA-seq techniques to explore the potential pathogenesis of KD and hypertrophic scars (24). Building on this approach, the present study further focuses on the mannose metabolic pathway, aiming to identify the specific cell types and subpopulations in which this pathway is aberrantly regulated, thereby elucidating its mechanistic role in the pathogenesis of KD at the cellular level.

By leveraging integrated bioinformatics analysis of public bulk and scRNA-seq datasets, we designed this study to identify and validate robust biomarkers related to mannose metabolism in KD. We employed differential expression analysis and advanced machine learning algorithms (e.g., Random Forest, Boruta) to screen for core genes associated with mannose metabolism and subsequently constructed a diagnostic nomogram. Furthermore, we utilized scRNA-seq data to resolve the cellular heterogeneity of KD and identify the key cell types responsible for the observed mannose metabolic signature. Our comprehensive approach aims to provide novel insights into KD pathogenesis and unveil potential diagnostic biomarkers and therapeutic targets, ultimately contributing to the development of more effective management strategies for this challenging cutaneous condition.

## 2 Materials and methods

### 2.1 Data source

The datasets GSE145725, GSE185309, and GSE163973 were downloaded from the Gene Expression Omnibus (GEO, https://www.ncbi.nlm.nih.gov/geo/). GSE145725 was derived from the GPL16043 platform, comprising 9 KD fibroblast samples from KD patients and 10 control fibroblast samples. The GSE185309 dataset was obtained from the GPL24676 genomic platform, comprising nine KD dermal tissue specimens alongside eight corresponding control dermal tissue specimens. The single-cell transcriptomic dataset GSE163973 was derived from the same GPL24676 platform, incorporating three KD tissue specimens and three matched control tissue specimens. Gene sets associated with mannose metabolic pathways were extracted from the Molecular Signatures Database (MSigDB, https://www.gsea-msigdb.org/gsea/msigdb/) through keyword-based searching utilizing the term “mannose”. A total of 11 relevant pathways were obtained, which were M3061, M29124, M29034, M29167, M16464, M12862, M17440, M18069, M15898, M47618, and M47610. After performing duplicate removal on the genes in these pathways, 160 mannose metabolism-related genes (MMRGs) were finally obtained (**Supplementary Table 1**).

### 2.2 Analysis of differential gene expression

The differentially expressed genes (DEGs) between KD and control samples in the GSE145725 dataset were identified using the “limma” package (v 3.58.1) (25)(p < 0.05, |log_2_ fold change (log_2_FC)| > 0.5). A volcano plot and a heatmap of DEGs were generated using the “ggplot2” package (v 3.5.1) (26) and the “ComplexHeatmap” package (v 2.10.8) (27). The figure was labelled the top 10 DEGs ranked in descending order of |log_2_FC|.

### 2.3 Functional analyses of candidate genes

For identifying KD-associated genes involved in mannose metabolic processes, the “ggvenn” bioinformatics package (28) was utilized to determine overlapping elements between differentially expressed genes and mannose metabolism-related genes, with these overlapping elements serving as candidate gene targets. Functional enrichment analyses encompassing Gene ontology (GO) and Kyoto Encyclopedia of Genes (KEGG) and Genomes pathway assessments were conducted on these candidate gene targets utilizing the “clusterProfiler” computational package (v 4.10.1) (28) with statistical significance threshold set at p < 0.05. The most statistically significant five results from both GO and KEGG enrichment analyses were presented in order of increasing p-value significance. To investigate protein-level interactions among candidate gene products, the Search Tool for Retrieval of Interacting Genes/Proteins database (STRING, http://www.string-db.org/) was employed for constructing comprehensive Protein-Protein Interaction network architectures. Furthermore, Cytoscape software (v 3.9.1) (29) was utilized for network visualization of candidate gene PPI networks, incorporating interactions with confidence threshold values exceeding 0.4.

### 2.4 Acquisition of biomarkers

In the GSE145725 dataset, a random forest (RF) model was constructed using the “randomForest” package (v 4.7.1.1) (30). Gene importance scoring for individual candidate genes was determined through mean decrease accuracy metrics, subsequently identifying the ten most significant genes based on their importance rankings. The Boruta feature selection algorithm was executed utilizing the “Boruta” computational package (v 8.0.0) (31). This algorithm generated a corresponding shadow feature for each real feature, and both the real features and shadow features were used together for random forest model training. On this basis, the importance Z-score based on the random forest was calculated. In each iteration, the algorithm compared the Z-value of each true feature with the maximum Z-value of all shadow features (shadowMax), and used a statistical test to determine whether the feature was significantly superior to the shadow features (pValue = 0.01, mcAdj = TRUE). The algorithm ran for a maximum of maxRuns = 200 iterations and retained the history (holdHistory = TRUE). Feature importance was calculated using the getImpRfZ function (which utilised the ranger package to implement standardised permutation-based importance, with ntree = 1000). The genes classified as “Confirmed” were the ones screened out by the Boruta algorithm. Subsequently, the “ggvenn” bioinformatics package (version 0.1.10) was utilized to identify overlapping gene elements between those identified through Random Forest methodology and Boruta feature selection algorithm, ultimately yielding the final characteristic gene signatures.

To investigate the expression of feature genes in the two datasets GSE145725 and GSE185309, the Wilcoxon test was used to analyse the expression of feature genes between KD samples and control samples in both datasets. Genes that showed significantly differential expression in both datasets with consistent expression trends were defined as candidate biomarkers (p < 0.05). To verify the diagnostic efficacy of candidate key biomarkers for KD, based on the KD and control samples in the GSE145725 and GSE185309 datasets, the “pROC” package (v 1.18.5) (32) was used to plot ROC curves for the candidate biomarkers and to calculate the Area Under Curve (AUC) values. Genes with an AUC > 0.7 were defined as biomarkers.

### 2.5 Construction and evaluation of the nomogram

For evaluating the prognostic performance of identified biomarkers in predicting KD development, a nomogram prediction model was developed utilizing the “rms” statistical package (33), incorporating biomarker expression profile data as foundational parameters. In this nomogram, corresponding scores were assigned to each biomarker, and the total score was calculated by summing individual scores. The prediction probability of outcome events for each individual was then derived through the functional relationship between total scores and the probability of outcome events. Subsequently, the “rms” package (v 6.8.1) was used to plot the model’s calibration curve (Hosmer-Lemeshow, p > 0.05), and the “ggDCA” package (v 1.1) (34) was utilized to create the decision curve analysis (DCA) curve (with the net benefit value greater than 0 as the criterion for good predictive performance). These analyses collectively validated the model’s predictive performance and clinical utility.

### 2.6 Gene set enrichment analysis

Utilizing KD and control specimen data derived from the GSE145725 genomic dataset, the reference gene collection “h.all.v2023.2.Hs.symbols” was obtained from the Molecular Signatures Database (MSigDB, https://www.gsea-msigdb.org/gsea/msigdb/). Correlation coefficient calculations between identified biomarkers and all remaining genomic elements were executed through the “psych” statistical package (v 2.4.3) (35), with subsequent gene ranking performed in descending order according to correlation strength values. Gene Set Enrichment Analysis was then conducted utilizing the “clusterProfiler” bioinformatics package (v 4.10.1) with filtering criteria including |Normalized Enrichment Score| (|NES|) exceeding 1.0, False Discovery Rate (FDR) below 0.25, and statistical significance threshold of p < 0.05. Visualised using the “enrichplot” graphical package. Furthermore, Spearman’s correlation analysis was performed using the psych statistical package (version 2.4.3) to examine the relationships between key genes and fibrosis-associated genes (including *TGFB1*, *COL1A1*, *FN1*, *ACTA2*, *SMAD3*, *CTGF*, *MMP2*, *TIMP1*, *IL1B* and *HSPA5*) (p < 0.05).

### 2.7 Chromosome localization

To investigate the chromosomal localization of the biomarkers, the “RCircos” package (v 1.2.2) (36) was used to annotate and plot gene localization maps, displaying the distribution of the biomarkers on chromosomes.

### 2.8 Compound prediction

To explore potential drugs or compounds that could target these biomarkers, the Drug Signature Database (DSigDB) (https://dsigdb.tanlab.org/DSigDBv1.0/) was applied to predict potential drugs or compounds associated with these biomarkers. The “enrichR” package (v 3.2) (37) was employed to perform association analysis between the screened biomarkers and the potential drugs or compounds. Additionally, the “ggplot2” package (v 3.5.1) was utilized to plot a sankey diagram illustrating the relationships between the biomarkers and the potential drugs or compounds.

### 2.9 Single-cell analysis

To obtain cells suitable for single-cell analysis, the “PercentageFeatureSet” function of the “Seurat” package (v 5.1.0) (38) was applied to perform strict quality control on KD and control samples in the GSE163973 dataset. Quality control parameters: Genomic elements detected in fewer than three cellular units were excluded from analysis; cellular specimens exhibiting suboptimal quality characteristics with gene detection below 200 transcripts were removed; cellular entities demonstrating exceptionally elevated transcriptomic activity (specifically, those with gene detection exceeding 6000 transcripts) were eliminated; cellular components displaying mitochondrial sequence read proportions above 20% threshold were excluded from downstream analysis; cells with total expression counts ≤ 500 or ≥ 10000 were filtered out. After the above filtering steps, the cells and genes that met the criteria were used for subsequent analyses. Following quality control procedures, the Harmony integration algorithm was employed to merge the processed single-cell transcriptomic datasets, while the “LogNormalize” computational function was utilized to achieve data normalization and standardization processes. Using the “Find Variable Features” function of the “Seurat” package (v 5.1.0), highly variable genes were identified among these cells based on the relationship between mean and variance, and the top 2000 genes with relatively large expression variations were recognized (selection.method = “vst”, nfeatures = 2000). The “LabelPoints” function visualized the results and annotated the top 10 genes with the highest expression variability. Data normalization procedures were executed through the “ScaleData” computational function within the “Seurat” bioinformatics package (v 5.1.0). Perform a principal component analysis (PCA) on the set of highly variable genes using “RunPCA”, and determine the number of principal components by combining the JackStraw test (num.replicate = 100, dims = 50) with the “Elbow plot.” The principal components (PCs) before the point where the plot tended to stabilize were selected for downstream analysis. To eliminate batch effects, data correction was performed using the Harmony algorithm. Subsequently, A k-nearest neighbours (k-NN) graph was constructed using the “FindNeighbours” function (k.param = 20, nn.method = “annoy”, n.trees = 50, prune.SNN = 1/15), followed by cell clustering using the “FindClusters” function, with a resolution of 0.8 and the default Louvain clustering algorithm (algorithm = 1). To visualize the clustering results, Uniform Manifold Approximation and Projection (UMAP) dimensionality reduction was performed using RunUMAP (umap.method = “uwot”, n.neighbors = 30, metric = “cosine”, min.dist = 0.3, seed.use = 42). Genes of various cell types were obtained from the literature (22). Bubble plot visualization of marker gene expression patterns across individual cellular clusters was generated utilizing the “DotPlot” graphical function within the “Seurat” bioinformatics package (v 5.1.0), with subsequent cellular phenotype identification accomplished through analysis of marker expression profiles. To display the proportion of various cell types in different samples, the “ggplot2” package (v 3.5.1) was used to plot cell proportion histograms.

### 2.10 Identification of key cells

To explore the biological pathways through which cells function, the “ReactomeGSA” package (v 1.16.1) (39) was used to perform functional enrichment analysis on each cell type separately, and the enrichment results were visualized. Subsequently, by reviewing relevant literature, the cell types that played a key role in the occurrence and development of KD were identified. Combined with the aforementioned functional enrichment analysis results, the key cells in the study were finally identified.

### 2.11 Cell communication, and pseudo-time series analysis

To clarify intercellular interactions, based on the KD and control samples in GSE163973, the “CellChat” package (v 1.6.1) (40) was used to analyse ligand-receptor (L-R) interactions between different cell types, following which a cell communication network was constructed. The communication probabilities of key ligand-receptor pairs between cell populations were calculated, and the results were visualized using bubble plots. To clarify the heterogeneity of key cells and their differentiation characteristics over time, the key cells were further divided into different subclusters (resolution = 0.1) according to the aforementioned dimensionality reduction and clustering methods. These subclusters were then annotated into corresponding cell types with reference to the marker genes provided in the literature (41). Following cellular clustering analysis, the “Monocle” computational package (v 2.28.0) (42) was employed to perform trajectory inference analysis on critical cellular populations, with subsequent assessment of biomarker dynamic expression profiles across distinct developmental stages within these critical cellular lineages.

### 2.12 Single-cell transcription factor analysis

To clarify the expression patterns of transcription factors at the single-cell level, using the expression matrices of each subpopulation of key cells as input, the two-step method of pySCENIC (GRNBoost2 to cisTarget) was followed for analysis: first, co-expression analysis was performed to infer candidate interaction edges between transcription factors and target genes, then motif enrichment and pruning were conducted using the hg38/mm10 motif/proximal site database, retaining only regulons supported by motifs to construct the Gene Regulatory Network (GRN). Subsequently, AUCell was used to calculate the AUC value of each regulon in every cell, and based on SCENIC’s (v 1.3.1) (43) regulon specificity score (RSS, based on Jensen-Shannon divergence), the specific activity scores of transcription factors in each subpopulation were evaluated. According to the ranking of these scores, the top 5 specific transcription factors in each subpopulation of key cells were screened out as key regulatory factors, and UMAP was used to visualize the expression of these key regulatory factors in key cells. After that, the target genes of the key regulatory factors were derived from the regulons pruned by cisTarget, and Cytoscape software (v 3.9.1) was used to construct regulatory networks between each subpopulation of key cells and their corresponding key regulatory factors.

### 2.13 Reverse transcription-quantitative polymerase chain reaction

Biomarker expression validation was accomplished through reverse transcription-quantitative polymerase chain reaction methodology. Five KD dermal tissue specimens alongside five control dermal tissue specimens were procured from GuangZhou Dermatology Hospital (gzsp202438) (**Supplementary Table 2**). All patients voluntarily underwent surgical excision and met the following inclusion criteria: age between 18 and 45 years; absence of local infection at the lesion site; no prior treatment with medication, radiation, or laser therapy; and no history of systemic organic disease. Healthy group with age and sex matched to the KD group; Excess normal skin tissue obtained during surgery for subcutaneous masses on the chest wall, back, or shoulder, confirmed by a dermatologist to be free of any skin lesions at the sampling site. Data visualization was performed utilizing Graphpad Prism software. Initially, ribonucleic acid isolation from the ten tissue specimens was conducted using TRIzol reagent (Novozymes, Nanjing, China) following established manufacturer protocols. Following RNA extraction, the HP All-in-one qRT Master Mix II RT203-Ver.1 reverse transcription kit (Yungene Bio, Kunming, China) was utilized to execute complementary DNA synthesis from total RNA templates, maintaining strict adherence to manufacturer specifications. Real-time quantitative PCR amplification was performed using 2×Universal Blue SYBR Green qPCR Master Mix reagent system (Servicebio, Wuhan, China). Polymerase chain reaction primer sequences are detailed in **Table 1**.

**Table 1:** Primer sequences and product lengths for MANBA, TMTC2, and GAPDH genes used in RT-qPCR validation.

Genes	Primer sequence	Product length
*MANBA* F	CTGGAGCATCTGCAATGGGA	233
*MANBA* R	AGCCTGACAAGTTGATGCCA	233
*TMTC2* F	GCATAGCACCTCTTTGACGGT	266
*TMTC2* R	GCTTCCCATGTCTCCCATGA	266
*H-GAPDH* F	ATGGGCAGCCGTTAGGAAAG	135
*H-GAPDH* R	AGGAAAAGCATCACCCGGAG	135

### 2.14 Statistical analysis

This study was analysed using R software (v 4.3.1). The Wilcoxon test was utilized to compare the differences between the two groups (p < 0.05).

## 3 Results

### 3.1 Enrichment analysis of 15 candidate genes

**Figure 1:**
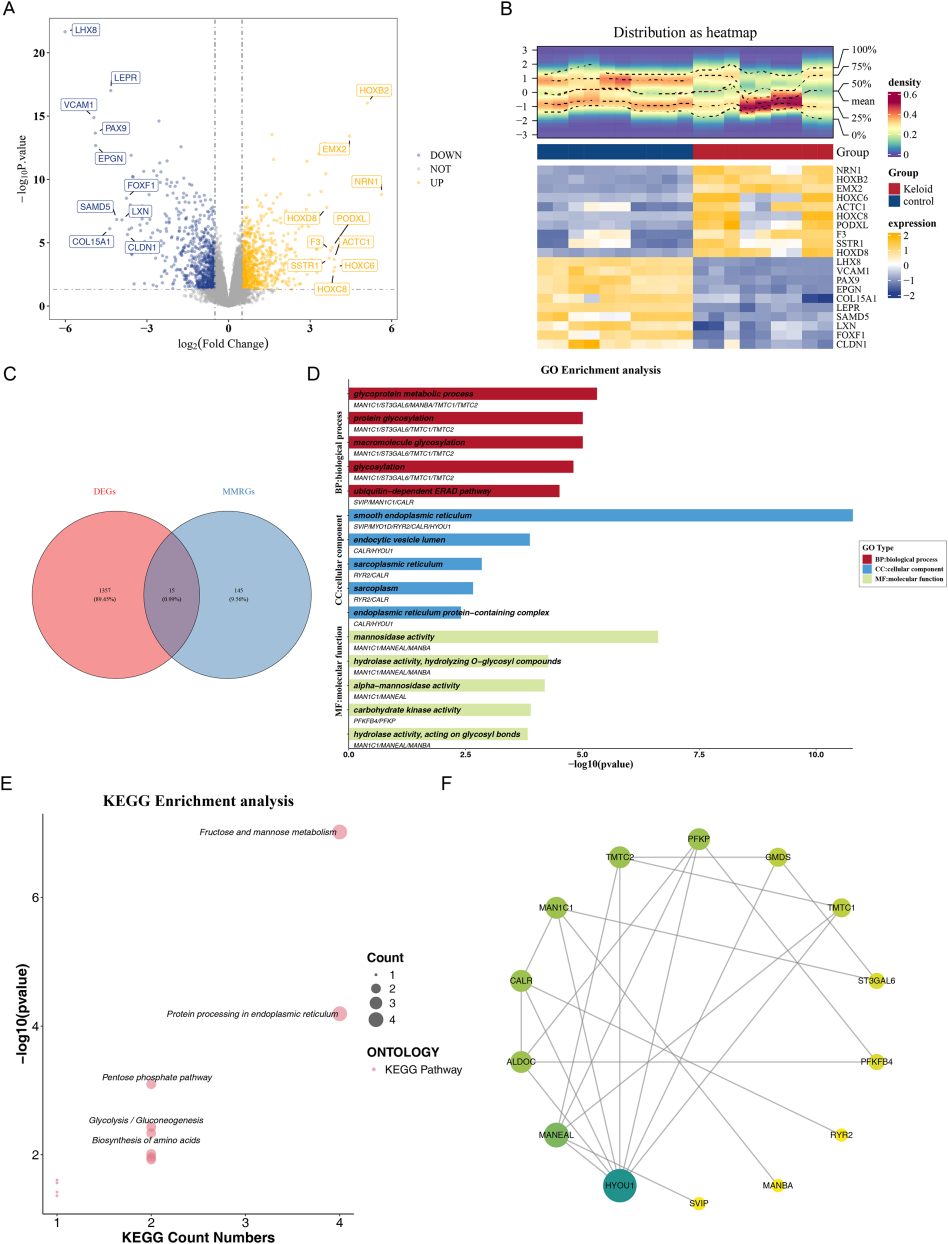
Identification and preliminary analysis of mannose metabolism-related candidate genes in KD. **(A)** Volcano plot displaying the 1,372 differentially expressed genes (DEGs) between KD and normal skin samples from the GSE145725 dataset. Yellow dots represent upregulated genes, blue dots represent downregulated genes (limma package; |log2FC|>0.5, p value<0.05). **(B)** Heatmap showing the expression patterns of the top DEGs across KD and control samples. The volcano plot displays the top 10 genes with the highest differential expression fold changes. The heatmap comprises two sections: the upper section presents a density heatmap of differential gene expression levels, illustrating five quantiles and a mean line; the lower section shows an expression heatmap for the differential genes. Colours represent gene expression magnitude, ranging from yellow to blue to indicate decreasing expression levels. **(C)** Venn diagram illustrating the intersection between 160 mannose metabolism-related genes (MMRGs) and the 1,372 DEGs, yielding 15 candidate genes. **(D)** Results of Gene Ontology (GO) enrichment analysis for the 15 candidate genes, showing the top significantly enriched terms in Biological Process (BP), Cellular Component (CC), and Molecular Function (MF) categories (p value<0.05). **(E)** Results of KEGG pathway enrichment analysis for the 15 candidate genes, showing the top significantly enriched pathways (p value<0.05). **(F)** Protein-protein interaction (PPI) network of the 15 candidate genes (confidence score > 0.4). Node size reflects the degree of interaction. Genes with the highest degree values (*HYOU1*, *MANEAL*, and *ALDOC*) are highlighted as potential hub genes. .

Differential gene analysis was performed on the KD samples and the control samples in the GSE145725 dataset, and a total of 1,372 DEGs were obtained. Among them, 672 DEGs were upregulated and 700 DEGs were downregulated in the KD samples (**Figures 1A, B**). Subsequently, after taking the intersection of 160 MMRGs and 1,372 DEGs, 15 candidate genes were obtained (**Figure 1C**). GO enrichment analysis revealed 231 significantly enriched terms, including 165 biological process (BP) terms, 26 cellular component (CC) terms, and 40 molecular function (MF) terms (**Supplementary Table 3**). More specifically, regarding biological processes classification, these genomic elements demonstrated predominant enrichment within glycoprotein metabolic pathways and protein glycosylation mechanisms. Concerning cellular component localization, these genes exhibited primary distribution patterns within smooth endoplasmic reticulum structures and endocytic vesicle luminal compartments. With respect to molecular function categorization, significant enrichment was observed in mannosidase enzymatic activity and hydrolase enzymatic functions involved in O-glycosyl compound hydrolysis reactions (**Figure 1D**). In the KEGG pathway analysis, 12 significantly enriched signalling pathways were obtained, such as fructose and mannose metabolism and protein processing in the endoplasmic reticulum (**Supplementary Table 4**, **Figure 1E**). The results of the PPI network showed that the three genes *HYOU1*, *MANEAL*, and *ALDOC* had the highest degree values in the network, which meant they interacted more frequently with other genes at the protein level. This indicated that these three genes might be the core hub genes in this network (**Figure 1F**).

### 3.2 *MANBA* and *TMTC2* were identified as potential biomarkers of KD

**Figure 2:**
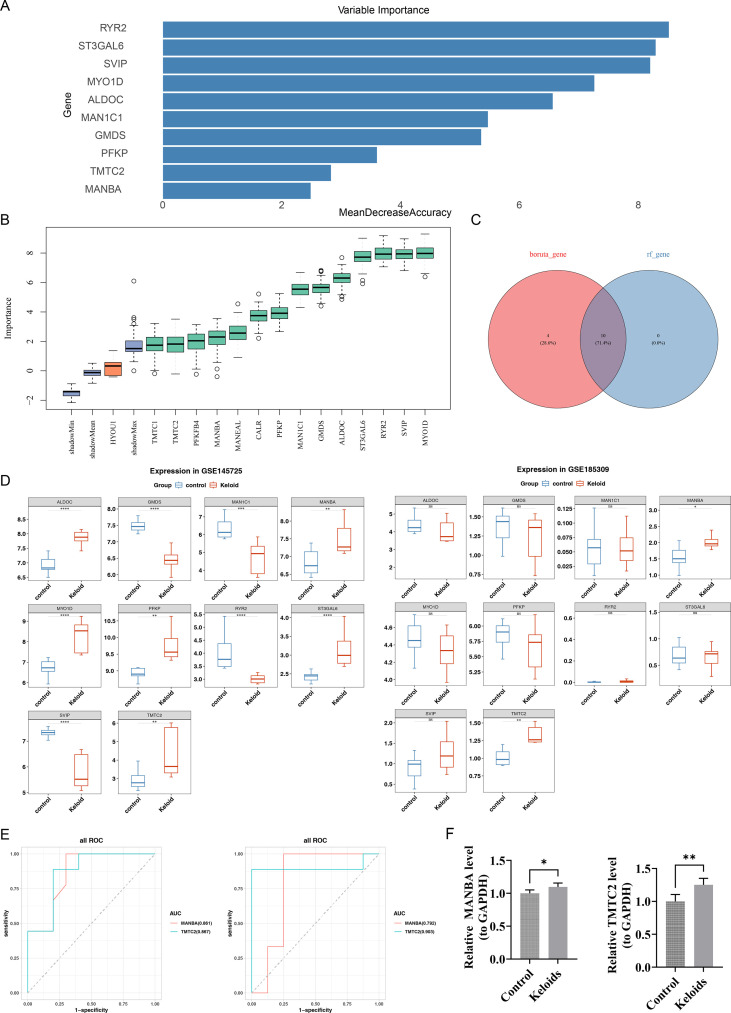
Screening and validation of biomarker genes for KD. **(A)** Bar plot of the importance scores of the top 10 candidate genes ranked by the Random Forest (RF) algorithm. Longer bars indicate greater significance. **(B)** Output plot of the Boruta algorithm showing the confirmation of 14 feature genes from the candidate list. Blue box plots correspond to shadow attributes, green box plots denote confirmed significant attributes, and orange box plots denote confirmed insignificant attributes. **(C)** Venn diagram identifying the 10 feature genes that were commonly selected by both the RF and Boruta algorithms. **(D)** Violin plots (or box plots) comparing the expression levels of the two final biomarker candidates, MANBA and TMTC2, between KD and control samples in the GSE145725 and GSE185309 datasets. Asterisks indicate statistical significance (Wilcoxon test, *p < 0.05). **(E)** Receiver Operating Characteristic (ROC) curves for *MANBA* and *TMTC2* in the GSE145725 and GSE185309 datasets (AUC > 0.7). The X-axis represents 1-specificity (false positive rate), while the Y-axis denotes sensitivity (true positive rate). The closer the AUC approaches 1, the better the predictive performance of the gene for the target feature. **(F)** Validation of *MANBA* and *TMTC2* expression by RT-qPCR in independent KD and control tissue samples. Data are presented as mean ± SEM, and statistical significance was determined by t-test (Graphpad Prism software, *p < 0.05, **p<0.01).

The feature genes were initially selected from candidate genes using the RF algorithm and the Boruta algorithm. Among them, the RF algorithm calculated the importance of each candidate gene and screened out the top 10 genes by importance (**Figure 2A**). Subsequently, the Boruta algorithm further screened and obtained 14 genes (**Figure 2B**). Ultimately, the intersection of genes selected by both algorithms was taken, resulting in 10 feature genes: *ST3GAL6*, *MYO1D*, *ALDOC*, *SVIP*, *RYR2*, *GMDS*, *MAN1C1*, *PFKP*, *MANBA*, and *TMTC2* (**Figure 2C**). The expression profiles of the 10 feature genes were analysed in KD samples versus control samples across the GSE145725 and GSE185309 datasets. The results demonstrated that both *MANBA* and *TMTC2* were significantly upregulated in KD samples compared to control samples, and this expression pattern was consistently observed in both datasets (p < 0.05). Based on these findings, *MANBA* and *TMTC2* were designated as candidate biomarkers for KD (**Figure 2D**). To further evaluate the discriminatory power of *MANBA* and *TMTC2* between KD and control samples, ROC analysis was performed. The results revealed that in both the GSE145725 and GSE185309 datasets, the AUC values for *MANBA* and *TMTC2* exceeded 0.7, indicating that these two genes possess robust diagnostic performance and could serve as reliable biomarkers for KD diagnosis (**Figure 2E**). Additionally, RT-qPCR results showed that *MANBA* and *TMTC2* genes were significantly upregulated in the KD group compared to the control group (p < 0.05) (**Figure 2F**). This finding was fully consistent with the bioinformatics analysis results, which indicated that the biomarkers identified through bioinformatics screening had reliable experimental validation.

### 3.3 Nomogram had good predictive performance

To evaluate the clinical value of the biomarkers in predicting the risk of KD onset, a nomogram prediction model was established. This predictive model was employed to generate scoring values for individual biomarkers, whereby each biomarker was assigned a unique numerical score; the aggregate scoring represented the summation of individual biomarker scores, with elevated aggregate scores correlating with increased disease development probability (**Supplementary Figure 1A**). The model calibration assessment revealed no statistically significant discrepancy between model-predicted probabilities and observed outcomes (p = 0.876), indicating that the predicted probability curve exhibited reasonable concordance with the observed probability curve (**Supplementary Figure 1B**). DCA revealed that the model exhibited high net benefit within the range of risk threshold probabilities, suggesting that the model had potential clinical predictive value (**Supplementary Figure 1C**). It should be emphasised that the above results were based solely on a limited sample size and internal validation; they had not been validated using independent external data. Consequently, the predictive performance of this model required further validation in future studies involving larger sample sizes and multicentre cohort studies.

### 3.4 Enrichment analysis of *MANBA* and *TMTC2* and compound prediction

**Figure 3:**
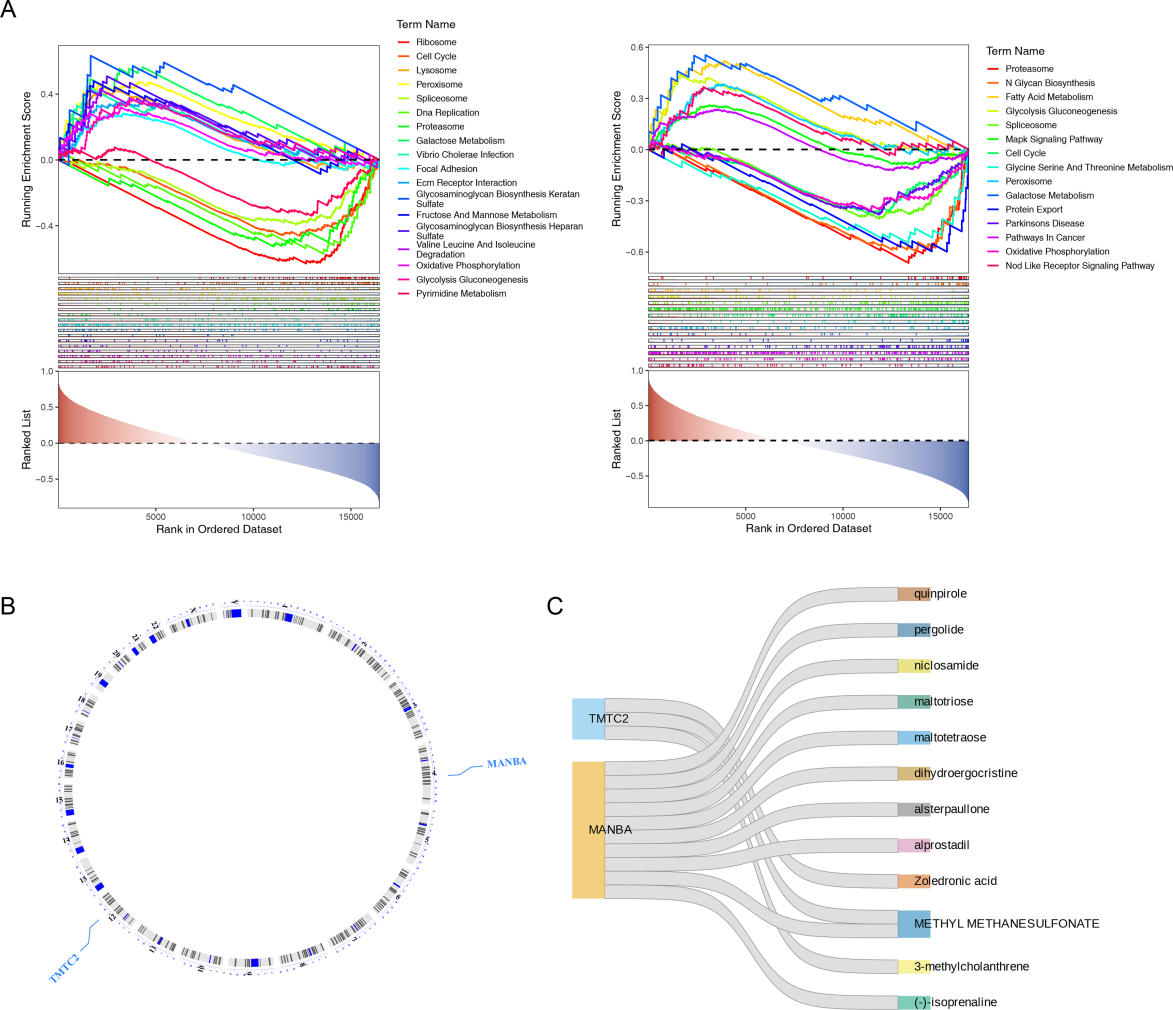
Functional enrichment, chromosomal localization, and potential drug prediction for *MANBA* and *TMTC2*. **(A)** Gene Set Enrichment Analysis (GSEA) results showing the top 10 significantly enriched pathways shared by *MANBA* and *TMTC2*, ranked by p-value (|NES| > 1.0, FDR < 0.25, and p < 0.05). Part 1 presents the Gene Enrichment Score, with the horizontal axis displaying the target gene set sorted by gene correlation. In this study, the region to the left of the peak represents genes positively correlated with the key gene, while the region to the right denotes genes negatively correlated with it. The vertical axis shows the corresponding Running ES. The peak of the line graph indicates the enrichment score for that pathway, with genes preceding the peak being core genes within that pathway from the tested gene set. Part 2 displays the hit, with lines marking genes located beneath the tested gene set. Part 3 presents the rank value distribution for all genes, using the Signal2Noise algorithm by default. **(B)** Schematic diagram showing the chromosomal locations of the *MANBA* (on chromosome 4) and *TMTC2* (on chromosome 12) genes. **(C)** Sankey diagram displaying the potential drug compounds associated with *MANBA* and *TMTC2* predicted from the DSigDB database. The left panel displays key genes, each associated with multiple compounds on the right. Each grey flowline indicates a potential relationship between a drug and its corresponding gene.

GSEA results demonstrated that *MANBA* and *TMTC2* were enriched in 18 and 15 pathways, respectively (**Figure 3A**, **Supplementary Tables 5, 6**). Both were significantly enriched in pathways such as the cell cycle, the proteasome and galactose metabolism. Notably, *MANBA* was significantly enriched in the ECM receptor interaction and focal adhesion pathways, which are highly correlated with the characteristics of excessive fibroblast adhesion and ECM deposition observed in KD. *TMTC2*, on the other hand, was significantly enriched in N-glycan biosynthesis, the MAPK signalling pathway and the NOD-like receptor signalling pathway, suggesting that it might participate in disease progression by regulating protein glycosylation, inflammatory responses and cell proliferation signals. It is worth noting that *MANBA* was significantly positively correlated with FN1, whilst *TMTC2* was significantly negatively correlated with *COL1A1* (p < 0.05) (**Supplementary Figure 2**). The results of the chromosome localization showed that *MANBA* and *TMTC2* were distributed on chromosome 4 and chromosome 12, respectively (**Figure 3B**). Subsequently, potential drug compounds associated with the biomarkers were predicted using DSigDB. Screening identified that *MANBA* was linked to 10 potential compounds, while *TMTC2* was associated with 3 compounds. Notably, methyl methanesulfonate emerged as a compound shared in association with both *MANBA* and *TMTC2* (**Figure 3C**).

### 3.5 Fibroblasts and keratinocytes were the key cells for KD

**Figure 4:**
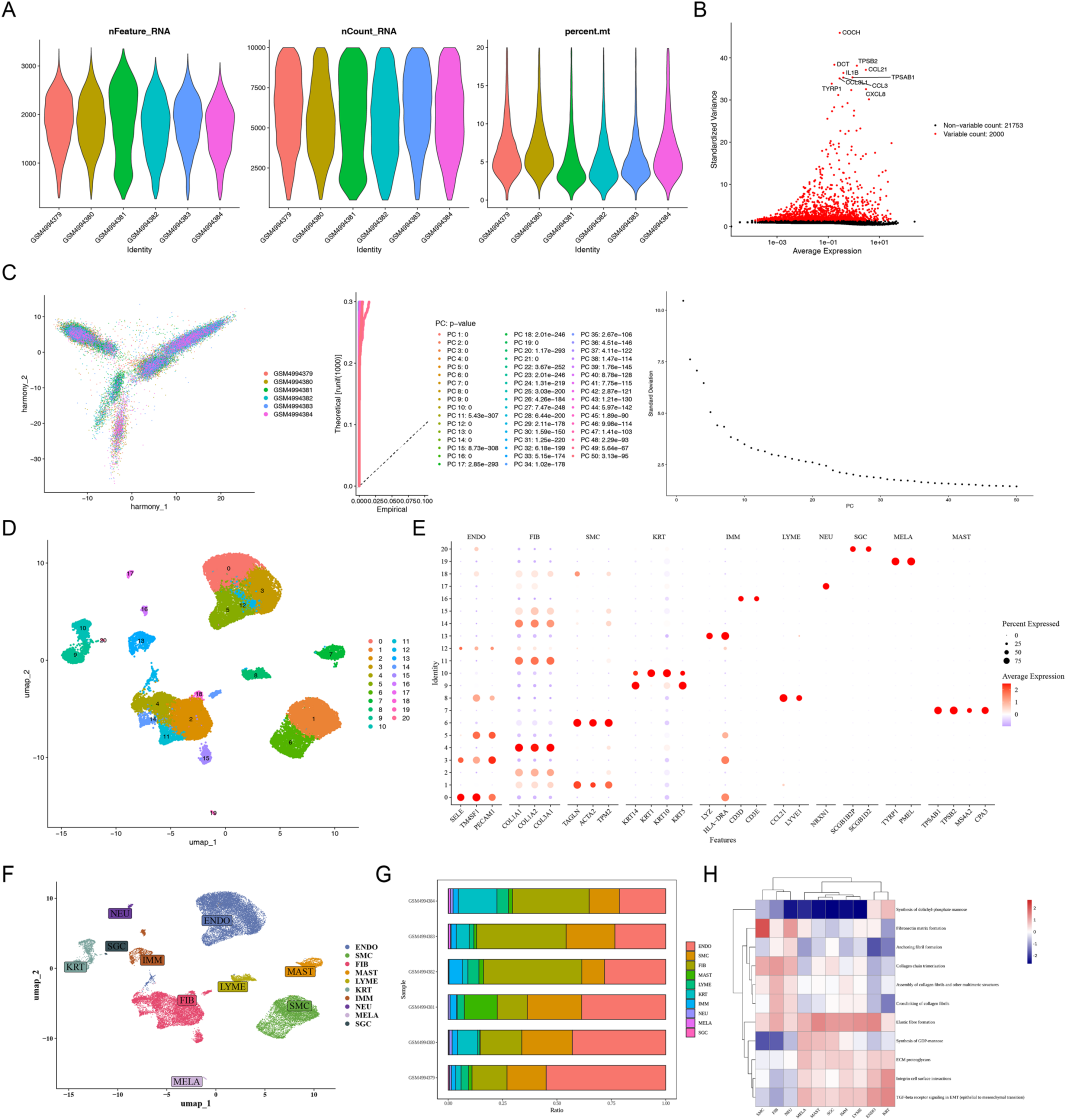
Single-cell RNA sequencing analysis identifies key cell types in KD. **(A)** Quality control (QC) metrics violin plots showing the distribution of detected genes, counts, and mitochondrial gene percentage per cell before and after filtering. Filtering criteria: Genes expressed in fewer than three cells; low-quality cells with fewer than 200 genes; abnormally highly expressed cells with more than 6,000 genes; cells with >20% mitochondrial reads were simultaneously excluded; cells with total expression levels ≤500 and ≥10,000 were removed. **(B)** Plot of highly variable genes identified in the scRNA-seq dataset. The top 10 most variable genes are labelled. **(C)** Scree plot of the principal components (PCs) showing the proportion of variance explained by each PC. The dotted line indicates the chosen cutoff (PC30) for downstream analysis. **(D)** UMAP plot visualizing 21 unsupervised cell clusters. **(E)** Marker gene expression bubble plots across clusters. The size of the bubble indicates the percentage of cells within a specific cell population expressing that gene, while the bubble’s colour represents the average expression level of that gene. A deeper red colour signifies a higher average expression level, and a larger bubble indicates greater expression coverage of that gene within the cell population. **(F)** UMAP plots annotating the 10 major cell types based on canonical marker genes: endothelial cells, smooth muscle cells, fibroblasts, mast cells, lymphatic endothelial cells, keratinocytes, immune cells, neural cells, melanocytes, and sweat gland cells. **(G)** Stacked bar plot showing the proportional abundance of each cell type across individual KD and control samples. **(H)** Bubble plot (or heatmap) of cellular functional enrichment analysis, displaying selected significantly enriched pathways for each cell type.

Comprehensive quality control procedures were executed on KD specimen data and control specimen data within the GSE163973 genomic dataset. Following quality control assessment, a total of 28,375 cellular entities and 23,753 genomic elements were preserved for downstream analytical procedures (**Figure 4A**). Following quality assessment, the leading 2,000 highly variable genomic elements were identified, with additional emphasis placed on the ten most variable genes demonstrating maximum variability characteristics (**Figure 4B**). Subsequently, principal component analysis methodology was employed to achieve dimensional reduction of the dataset; based on the inflection point coordinates within the scree plot visualization, the leading 30 principal components were selected for further analytical processes (**Figure 4C**). Cellular clustering analysis was performed utilizing the Uniform Manifold Approximation and Projection algorithm, resulting in the identification of 21 distinct cellular cluster populations (**Figure 4D**). Finally, marker genes (**Table 2**) for each cell type were obtained based on existing literature, and 10 cell types were identified accordingly, which were endothelial cells, smooth muscle cells, fibroblasts, mast cells, lymphatic endothelial cells, keratinocytes, immune cells (IMM), neural cells, melanocytes, and sweat gland cells (**Figures 4E, F**). After analysing the proportion of each cell type among different samples, it was found that the proportions of endothelial cells, smooth muscle cells, and fibroblasts were relatively high in all samples (**Figure 4G**). Cellular functional enrichment analysis revealed that smooth muscle cells and neural cells were primarily enriched in the “fibronectin matrix formation” pathway. Fibroblasts were shown in multiple pathways, including “collagen chain trimerization” and “elastic fibre formation”. Melanocytes were mainly enriched in pathways such as “elastic fibre formation” and “synthesis of GDP-mannose”. Mast cells, sweat gland cells, immune cells, lymphatic endothelial cells, and endothelial cells were significantly enriched in the “elastic fibre formation” pathway. Keratinocytes were primarily enriched in pathways including “integrin cell surface interactions” and “TGF-beta receptor signalling in EMT (epithelial to mesenchymal transition)” (**Figure 4H**). Finally, through literature review, it was found that fibroblasts and keratinocytes were relatively important cells in the occurrence and development of KD; further combining their functions, the two cell types were identified as the key cells of KD.

**Table 2:** Marker genes used for the identification of major cell types in scRNA-seq analysis.

Cell type	Marker
endothelial cells	*SELE, TM4SF1, PECAM1*
fibroblasts	*COL1A1, COL1A2, COL3A1*
smooth muscle cells	*TAGLN, ACTA2, TPM2*
keratinocytes	*KRT14, KRT1, KRT10, KRT5*
immune cells	*LYZ, HLA-DRA, CD3D, CD3E*
lymphatic endothelial cells	*CCL21, LYVE1*
neural cells	*NRXN1*
sweat gland cells	*SCGB1B2P, SCGB1D2*
melanocytes	*TYRP1, PMEL*
mast cells	*TPSAB1, TPSB2, MS4A2, CPA3*

### 3.6 Communication networks of key cells

**Figure 5:**
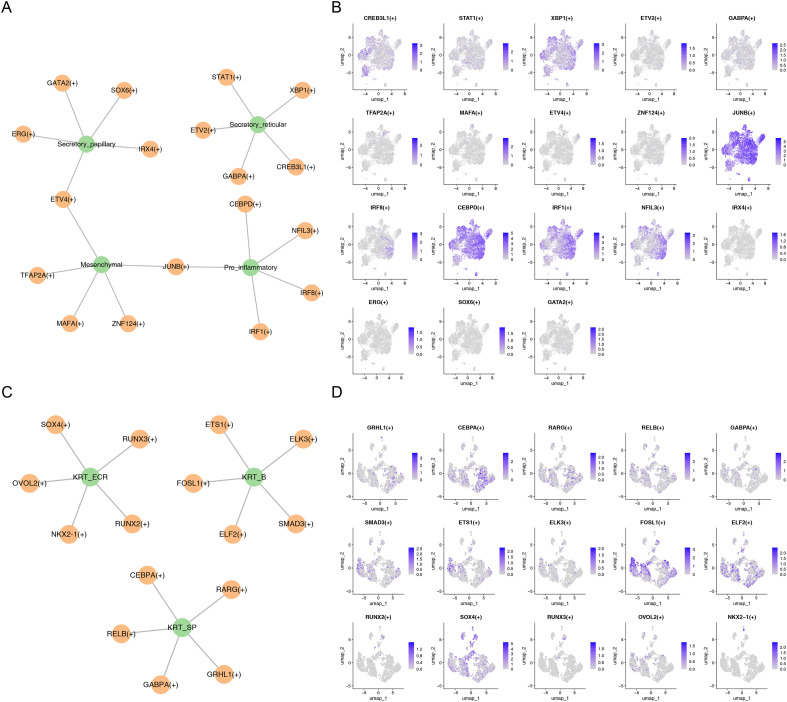
Analysis of cell-cell communication networks in KD versus normal skin. **(A, B)** The cellular communication network diagram illustrates the quantity and strength of ligand-receptor interactions between different cell types in the control group **(A)** and the KD group **(B)**. Different colours denote different cell types, while the thickness of the edges indicates the volume/intensity of communication. **(C)** Bubble chart showing the top ligand-receptor pairs for fibroblasts when acting as signal senders (ligands) or receivers (receptors) in control and KD groups. **(D)** Bubble chart showing the top ligand-receptor pairs for keratinocytes when acting as signal senders (ligands) or receivers (receptors) in control and KD groups. Each dot represents a ligand-receptor pair between specific cell types. The dot size indicates the significance of the interaction (p-value), while the colour denotes communication strength (Comm. Prob), ranging from blue to red to represent progressively higher communication probabilities. The horizontal axis shows cell type pairings, and the vertical axis displays ligand-receptor pairs.

Cell-cell communication analysis revealed widespread interactions among most cell types. Among them, compared with the control group, the number of interactions between smooth muscle cells and most cell types, including fibroblasts, lymphatic endothelial cells, and keratinocytes, increased; notably, both the number and intensity of interactions between the key cell fibroblasts and smooth muscle cells, keratinocytes, immune cells, and neural cells increased (**Figures 5A, B**). When Fibroblasts were used as ligands or receptors, in the control group, the ligand-receptor pairs with the highest communication probability were CXCL2-ACKR1 and PTN-NCL, respectively; while in the KD group, the ligand-receptor pairs with the highest communication probability were both PTN-NCL (**Figure 5C**). When Keratinocytes were used as ligands or receptors, the ligand-receptor pairs with the highest communication probability showed consistency between the control group and the KD group, which were MIF-(CD74+CD44) and PTN-NCL, respectively (**Figure 5D**). This result suggested that PTN-NCL might be the core ligand-receptor pair for communication between Fibroblasts, Keratinocytes, and other cells.

### 3.7 Pseudo-temporal analysis of fibroblasts and keratinocytes

**Figure 6:**
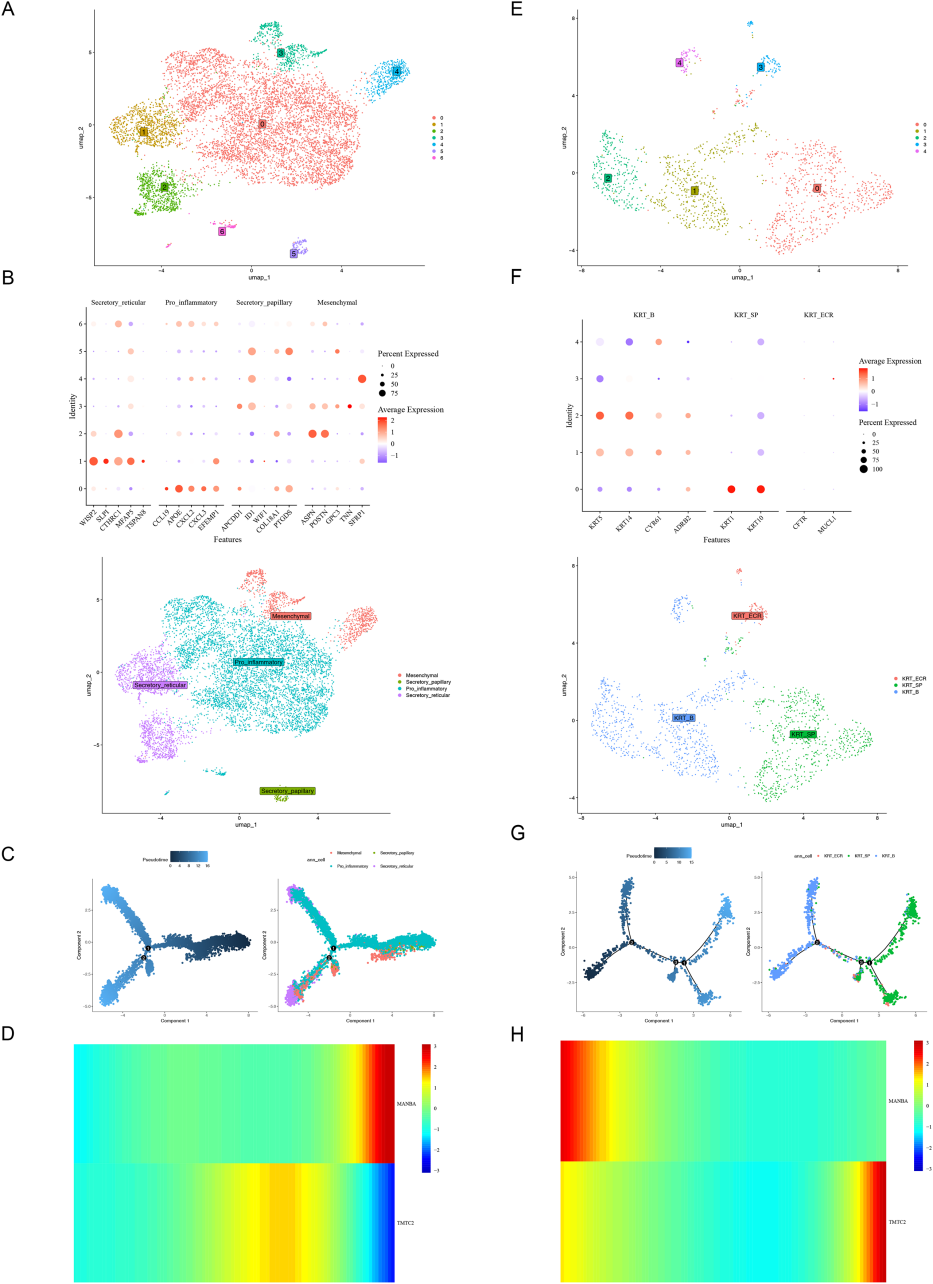
Pseudotemporal trajectory analysis of fibroblast and keratinocyte subpopulations. **(A)** UMAP plot showing the re-clustering of fibroblasts into 7 subclusters. **(B)** Annotation of the 4 major fibroblast subtypes: Secretary papillary, Pro-inflammatory, Secretary reticular, and Mesenchymal, based on marker genes. **(C)** Pseudotime trajectory analysis of fibroblasts. Cells are colored by their inferred developmental pseudotime. Colours ranging from light to dark denote different stages of pseudotime. Pseudotemporal analysis reveals the trajectories of cells during development or state transitions, wherein each node and branch represents distinct developmental phases or differentiation pathways of cellular states. **(D)** Heatmap plots showing the dynamic expression changes of *MANBA* and *TMTC2* along the fibroblast pseudotime trajectory. **(E)** UMAP plot showing the re-clustering of keratinocytes into 5 subclusters. **(F)** Annotation of the 3 major keratinocyte subtypes: KRT_B, KRT_SP, and KRT_ECR. **(G)** Pseudotime trajectory analysis of keratinocytes. Cells are colored by their inferred developmental pseudotime. **(H)** Heatmap plots showing the dynamic expression changes of *MANBA* and *TMTC2* along the keratinocyte pseudotime trajectory.

Fibroblasts were subjected to secondary clustering, and a total of 7 cell clusters were identified (**Figure 6A**). Based on previously reported marker genes (**Table 3**) in the literature, these clusters were annotated as Secretory papillary, Pro-inflammatory, Secretory reticular, and Mesenchymal (**Figure 6B**). During the differentiation of Fibroblasts, the cell colour intensity showed a significant correlation with the developmental stage, with darker colours representing earlier developmental stages: among them, Secretory papillary was mainly distributed in the early and middle stages of differentiation, Pro inflammatory was distributed in the early, middle, and late stages of differentiation, Secretory reticular was concentrated in the late stage of differentiation, and Mesenchymal mainly appeared in the middle and late stages of differentiation (**Figure 6C**). Additionally, *TMTC2* expression exhibited a trend of initial increase followed by a decrease during fibroblast differentiation, while *MANBA* expression continuously increased with differentiation progression (**Figure 6D**). Keratinocytes were also subjected to secondary clustering, and 5 cell clusters were identified (**Figure 6E**). Based on characteristic marker genes (**Table 4**) from the literature, these clusters were annotated as KRT_B, KRT_SP, and KRT_ECR types (**Figure 6F**). Based on characteristic marker genes from the literature, these clusters were annotated as KRT B, KRT SP, and KRT ECR types. During the differentiation of Keratinocytes, there were differences in the stage distribution of cell clusters: KRT B was mainly distributed in the early and middle stages of differentiation, while KRT SP and KRT ECR were mainly concentrated in the middle and late stages of differentiation (**Figure 6G**). Regarding gene expression changes, *MANBA* expression gradually decreased with keratinocyte differentiation progression, whereas *TMTC2* expression exhibited a trend of initial decrease followed by an increase (**Figure 6H**). This indicated that the differentiation processes of fibroblasts and keratinocytes had stage-specificity, and the expression trends of the biomarkers *TMTC2* and *MANBA* showed differential regulation depending on cell types and differentiation stages.

**Table 3:** Marker genes used for the subclustering annotation of fibroblast subtypes.

Cell type	Marker
Secretory_reticular	*WISP2, SLPI, CTHRC1, MFAP5, TSPAN8*
Pro_inflammatory	*CCL19, APOE, CXCL2, CXCL3, EFEMP1*
Secretory_papillary	*APCDD1, ID1, WIF1, COL18A1, PTGDS*
Mesenchymal	*ASPN, POSTN, GPC3, TNN, SFRP1*

**Table 4:** Marker genes used for the subclustering annotation of keratinocyte subtypes.

Cell type	Marker
KRT_B	*KRT5, KRT14, CYR61, ADRB2*
KRT_SP	*KRT1, KRT10*
KRT_ECR	*CFTR, MUCL1*

### 3.8 Single-cell transcription factor regulatory network

**Figure 7:**
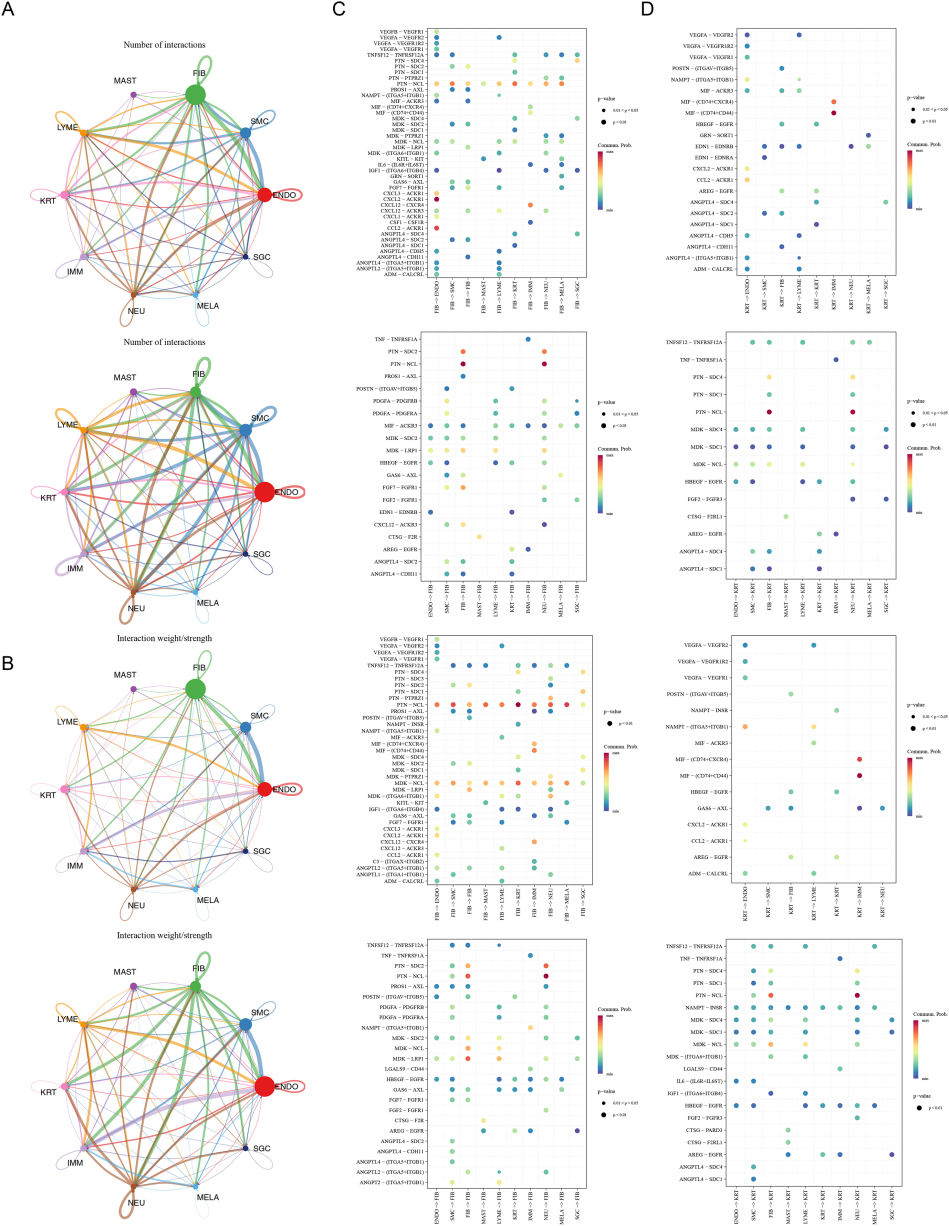
Transcription factor regulatory network analysis in fibroblast and keratinocyte subpopulations. **(A)** Network diagram of fibroblast subpopulations and key regulatory factors.The color and size of the dots represent the average expression level and the percentage of cells expressing the TF, respectively. ‘+’ indicates activation. **(B)** UMAP plot visualizing the expression distribution of representative highly expressed TFs (e.g., *JUNB*, *CEBPD*, *IRF1*) across all fibroblast subclusters. The bluer the colour, the higher the expression level. **(C)** Network diagram of keratinocyte subtypes and key regulatory factors. **(D)** UMAP plot visualizing the expression distribution of a representative highly expressed TF (*FOSL1*) across all keratinocyte subpopulations. The bluer the colour, the higher the expression level.

The top 5 specific transcription factors for each fibroblast subtype were as follows: the secretory reticular subtype included *CREB3L1* (+), *STAT1* (+), *XBP1* (+), *ETV2* (+), and *GABPA* (+); the secretory papillary subtype included *ETV4* (+), *IRX4* (+), *ERG* (+), *SOX6* (+), and *GATA2* (+); the pro-inflammatory subtype included *IRF8* (+), *CEBPD* (+), *IRF1* (+), *JUNB* (+), and *NFIL3* (+); the mesenchymal subtype included *TFAP2A* (+), *MAFA* (+), *ETV4* (+), *ZNF124* (+), and *JUNB* (+) (**Figure 7A**). Expression analysis revealed that transcription factors such as *JUNB* (+), *CEBPD* (+), and *IRF1* (+) exhibited relatively high expression levels across multiple fibroblast subclusters (**Figure 7B**). The top 5 specific transcription factors for each subpopulation of keratinocytes were respectively: KRT B subtype included *SMAD3* (+), *ETS1* (+), *ELK3* (+), *FOSL1* (+), and *ELF2* (+); KRT S Psubtype included *GRHL1* (+), *CEBPA* (+), *RARG* (+), *RELB* (+), and *GABPA* (+); KRT ECR subtype included *RUNX2* (+), *SOX4* (+), *RUNX3* (+), *OVOL2* (+), and *NKX2*-1 (+) (**Figure 7C**). Among these factors, *FOSL1* (+) exhibited relatively high expression levels in each subpopulation of keratinocytes (**Figure 7D**). This indicated that these highly expressed transcription factors might play a crucial regulatory role in the pathological process of KD.

## 4 Discussion

KD represents a significant challenge in dermatology, defined as a pathological fibroproliferative disorder of the skin. The primary issue with KD is their aggressive and invasive accumulation of collagen and ECM, the scope of which extends well beyond the boundaries of the original wound. This abnormal healing process is now recognized as arising from a complex interplay of genetic factors, persistent immune dysregulation, and severely altered cellular behaviours, all of which contribute to the difficulty in treating these lesions (4, 5, 7, 44). Mannose, a crucial hexose sugar, functions not merely in basic metabolic processes; it instead plays key roles in protein glycosylation and various specialized biosynthetic pathways (e.g., glycoconjugate synthesis). Accumulating evidence suggests that mannose metabolism may exert a substantial impact on critical pathological processes in KD, such as the excessive proliferation of fibroblasts, prolonged inflammatory responses, and severely disrupted fibrotic processes. This suggests that targeting mannose metabolism could provide a novel and promising therapeutic strategy (17, 18). However, the specific molecular mechanisms linking mannose metabolic pathways to KD development remain largely unelucidated and demand systematic exploration to identify potential therapeutic targets.

To elucidate the unclear molecular association between mannose metabolism and the pathogenesis of KD, as mentioned earlier, this study employed an integrated multi-omics strategy, combining bulk tissue RNA-seq and scRNA-seq data, aiming to screen and validate KD potential biomarkers related to mannose metabolism. The research first identified candidate genes by intersecting DEGs between KD and normal skin tissues with a carefully curated set of MMRGs. These candidate genes were subsequently refined using machine learning approaches and experimentally validated for their expression levels and diagnostic value. The workflow ultimately identified two core biomarkers—*MANBA* and *TMTC2*—which were consistently upregulated across different KD cohorts, suggesting their potential relevance to kD pathogenesis. A nomogram constructed based on these two biomarkers demonstrated promising potential for the clinical diagnosis of KD. Furthermore, scRNA-seq analysis elucidated the cellular heterogeneity within KD tissues, identifying fibroblasts and keratinocytes as key cell types driving disease progression. Pseudotime trajectory analysis revealed that *MANBA* and *TMTC2* exhibited cell type-specific and stage-dependent expression patterns during the differentiation of these critical cells. This suggests a possible role for these biomarkers in KD development and progression, potentially through involvement in cell differentiation and intercellular communication.

The *MANBA* gene encodes the enzyme β-mannosidase, a key component of the glycosyl hydrolase 2 family that is predominantly localized to lysosomes. This enzyme acts as a vital exoglycosidase, mediating the final stages of the catabolic process for N-linked glycoprotein oligosaccharides. Consequently, the protein encoded by *MANBA* is essential for maintaining intracellular homeostasis and metabolic balance through effective glycoprotein processing and turnover. Mutations that result in the loss of function of *MANBA*, leading to a marked reduction in β-mannosidase activity, are implicated in β-mannosidosis. This rare yet severe lysosomal storage disorder is characterized by substantial neurological deficits, underscoring the indispensable and non-replaceable physiological function of this gene in cellular homeostasis (45–47). Previous studies have identified an association between polymorphisms in the *MANBA* gene and the risk of cutaneous melanoma (48), suggesting that this gene may have broader pathophysiological implications in skin-related disorders. Based on this, the present study further focuses on its role in KD. Through bioinformatics analysis, we found that *MANBA* is significantly enriched in signalling pathways closely associated with fibrosis, such as ECM–receptor interaction and focal adhesion (49). Given that *MANBA* encodes β-mannosidase, a key enzyme in glycoprotein metabolism, and that the extracellular matrix itself is a dynamic network composed of proteins and glycosaminoglycans (50), we hypothesize that *MANBA* may participate in maintaining the dynamic balance between matrix synthesis and degradation by regulating the metabolism of ECM glycoproteins. Elevated *MANBA* expression may thereby promote excessive collagen deposition and the formation of a fibrotic microenvironment. Single-cell analyses further support this hypothesis, revealing that *MANBA* expression increases progressively throughout fibroblast differentiation. Notably, the secretory reticular subpopulation is predominantly enriched at the late differentiation stage, while the interstitial subpopulation is concentrated at the mid-to-late stages; both subsets typically exhibit enhanced ECM synthetic capacity and profibrotic activity (22, 51). As fibroblast differentiation progresses, *MANBA* expression continues to rise, suggesting that it may provide metabolic support for the synthesis of matrix proteins such as collagen, thereby sustaining sustained fibroblast activation and establishing a profibrotic positive feedback loop.

By contrast, *TMTC2*—another key biomarker identified in our study—encodes an integral membrane protein localized to the endoplasmic reticulum. It contains multiple tetratricopeptide repeat motifs, which are critical for mediating specific protein-protein interactions and forming multi-protein complexes. Emerging studies have demonstrated that the TMTC2 protein physically interacts with both sarco/endoplasmic reticulum calcium ATPase 2b (SERCA2b) and the carbohydrate-binding chaperone calnexin, thereby playing an essential role in maintaining calcium ion homeostasis within the ER lumen. This process is critically important for ensuring proper protein folding, post-translational modifications, and overall cellular functional integrity (52, 53). In this study, GSEA analysis revealed that *TMTC2* is primarily enriched in the NOD-like receptor signalling pathway and the MAPK signalling pathway. Among these, activation of NOD-like receptors, particularly the NLRP3 inflammasome, is closely associated with endoplasmic reticulum stress and calcium homeostasis imbalance (54, 55). Upon inflammasome activation, the release of IL-1β and IL-18 acts as potent pro-inflammatory factors, recruiting immune cells and activating fibroblasts, thereby serving as critical initiators of fibrosis (56). Meanwhile, activation KD of the MAPK pathway promotes fibroblast proliferation, differentiation into myofibroblasts, and stimulates the synthesis of extracellular matrix components such as collagen (57, 58). Additionally, calcium signalling influences multiple aspects of fibroblast activity. Therefore, we hypothesize that aberrant expression of *TMTC2* in s may contribute to disease progression by disrupting endoplasmic reticulum calcium homeostasis, thereby coordinately regulating NOD-like receptor-mediated inflammatory responses and MAPK pathway-mediated fibrotic effects. However, these mechanistic inferences are primarily based on bioinformatics analyses and literature support. The specific regulatory targets of *MANBA* in ECM glycoprotein metabolism and its causal relationship with fibroblast activation subpopulations, as well as the molecular mechanisms by which *TMTC2* modulates inflammatory signalling and fibrotic pathways through calcium homeostasis, remain to be further validated through *in vitro* cellular experiments and *in vivo* animal models.

In this study, RT-qPCR validated a significant upregulation of *MANBA* and *TMTC2* mRNA in KD tissues (*p* < 0.05), which is highly consistent with the multi-omics bioinformatics analyses, thereby enhancing the credibility of these two genes as KD-associated potential biomarkers. Unlike traditional fibrotic markers such as collagen and TGF-β1, which primarily reflect end-stage disease effects, *MANBA* and *TMTC2* are involved in mannose metabolism and endoplasmic reticulum calcium homeostasis, respectively, suggesting that they may represent upstream metabolic reprogramming events during disease initiation and progression. This metabolic aberration not only provides a novel perspective on the pathogenesis of KD but also confers dual value on these two genes as both diagnostic potential biomarkers and potential therapeutic targets. Targeting metabolic pathways may inhibit excessive fibroblast activation at the source. Future studies could explore the combined application of *MANBA* and *TMTC2* with existing clinical markers to further improve diagnostic accuracy and risk stratification for KD. Additionally, loss- and gain-of-function experiments at the cellular level should be conducted to clarify the direct effects of *MANBA* and *TMTC2* on fibroblast proliferation, collagen synthesis, and keratinocyte function.

Single-cell analysis revealed that fibroblasts and keratinocytes are key cell types involved in the initiation and progression of KD. In KD lesions, fibroblasts exhibit marked hyperactivation, characterized by aberrant proliferation and excessive synthesis and secretion of extracellular matrix components, which are considered core drivers of abnormal KD tissue proliferation (59). This finding is consistent with our observation that fibroblasts are significantly enriched in fibrosis-related pathways such as “collagen chain trimerization” and “elastic fibre formation”. To further dissect their heterogeneity, we performed subpopulation analysis of fibroblasts and annotated them into four subsets: secretory papillary, pro-inflammatory, secretory reticular, and mesenchymal fibroblasts. Notably, previous studies have reported that the proportion of the mesenchymal fibroblast subset is significantly increased in KD tissues compared with normal scars (22). Further insights from cancer-associated fibroblast studies have shown that cancer-associated fibroblasts can be divided into subsets with distinct functional characteristics, involving pro-inflammatory, pro-fibrotic, and iron metabolism-regulating functions (60). The pro-inflammatory and mesenchymal fibroblast subsets identified in our study share certain similarities in transcriptional features and functional tendencies with specific subsets of cancer-associated fibroblasts described above, suggesting that certain pathogenic fibroblast subsets may be shared across different fibroproliferative disorders.

Further cell–cell communication analysis revealed that the PTN-NCL ligand–receptor pair plays a central role in the communication between fibroblasts and keratinocytes in KD, suggesting that this signalling axis may serve as a key molecular hub driving the fibrotic process in KD. PTN, a highly conserved secreted ECM-associated protein, has been reported to be closely associated with the pathogenesis of hypertrophic scars (61). Studies have shown that in fibroblasts, PTN expression is regulated in a JUN-dependent manner; it not only promotes keratinocyte proliferation but is also further upregulated when co-cultured with keratinocytes. In turn, keratinocytes enhance PTN expression in fibroblasts, thereby forming a potential bidirectional positive feedback regulatory network (62). More notably, a recent study in the neurofibroma microenvironment revealed that Schwann cell-derived PTN acts in a paracrine manner on the NCL receptor on the surface of fibroblasts, activating the PRAS40 signalling molecule, significantly promoting fibroblast proliferation and collagen synthesis, and synergizing with TGF-β1 to enhance fibrotic effects (63). Based on the above evidence, we hypothesize that the PTN-NCL signalling axis may establish a pro-fibrotic positive feedback regulatory network between fibroblasts and keratinocytes in KD through similar paracrine and autocrine mechanisms. However, the present study is primarily based on bioinformatics predictions and expression validation, and the specific regulatory mechanisms have not yet been thoroughly elucidated. Future studies should employ *in vitro* co-culture models and *in vivo* functional experiments to further validate the functional role of the PTN-NCL axis in KD and to explore its synergistic mechanisms with classical fibrotic pathways such as TGF-β1.

Furthermore, in the pro-inflammatory fibroblast subset, we observed high expression of *JUNB* and *IRF1*, both of which are key regulators of inflammatory processes (64, 65). It has been well established that inflammation is often accompanied by a metabolic shift from oxidative phosphorylation to aerobic glycolysis (66, 67); in fibroblasts located in hypoxic regions of KD, glycolysis-related markers have also been shown to be upregulated (68). Mannose, an epimer of glucose, shares closely linked metabolic pathways with glycolysis and can be phosphorylated by hexokinase to enter glycolysis or glycosylation pathways (69). Based on this, we hypothesize that the inflammatory response driven by *JUNB* and *IRF1* may indirectly influence mannose metabolism by enhancing glycolysis. Meanwhile, mannose can also reciprocally regulate inflammatory processes (70), suggesting a potential bidirectional regulatory mechanism between the two. This hypothesis provides a potential upstream regulatory perspective for understanding aberrant mannose metabolism in KD, although the specific mechanisms remain to be validated by future functional experiments.

This study has several limitations. First, due to constraints in public database resources, the transcriptome datasets used in this study had relatively small sample sizes, which may affect the statistical power and stability of the results during the screening phase. Moreover, the sample size of the subsequent validation cohort was also limited (only five pairs of tissues), further restricting the generalizability and robustness of the conclusions. Second, the nomogram prediction model constructed based on *MANBA* and *TMTC2* was not validated using an independent external cohort, and the limited sample size used for model development poses a potential risk of overfitting. Additionally, although bioinformatics analyses suggested *MANBA* and *TMTC2* as potential biomarkers and implicated pathways such as the PTN-NCL cell interaction axis and cell cycle, the study lacks *in vitro* and *in vivo* functional validation, and the association between these genes and KD cannot be inferred as causal. Finally, the compounds predicted based on the DSigDB database (such as methyl methanesulfonate) only provide preliminary clues and lack experimental or clinical data support. To address these limitations, future studies will expand the sample size through collaboration with multicentre hospitals to further investigate the diagnostic value of *MANBA* and *TMTC2* and their correlation with clinical characteristics, and validate their expression at the protein level using Western blot and immunofluorescence assays. Additionally, we will establish cell and animal models and perform loss- or gain-of-function experiments using siRNA or CRISPR technologies to systematically evaluate the effects of *MANBA* and *TMTC2* on fibroblast proliferation, migration, collagen synthesis, and key signalling pathways (e.g., TGF-β, NF-κB). Combined with immunofluorescence co-localization and co-culture systems, we will further dissect the specific roles of intercellular communication, such as the PTN-NCL axis, in the KD microenvironment. Furthermore, dose-response functional experiments will be conducted to validate the effects of the predicted compounds on fibroblast activation or collagen synthesis, providing experimental evidence for the exploration of targeted therapeutic strategies.

## 5 Conclusion

Based on bioinformatics approaches, this study identified two genes associated with mannose metabolism, *MANBA* and *TMTC2*. Both genes were upregulated in KD tissues and may be involved in the regulation of pathways such as NOD-like receptor signalling, ECM–receptor interaction, and galactose metabolism, suggesting their potential as diagnostic biomarkers. Further analysis indicated that these two genes may be associated with the differentiation processes of fibroblasts and keratinocytes. In addition, intercellular communication analysis suggested that the PTN-NCL ligand–receptor pair may play a role in the regulation of the KD microenvironment. These findings provide new insights into the potential role of mannose metabolism in the initiation and progression of KD and lay a foundation for subsequent functional studies and the exploration of therapeutic targets. However, the conclusions of this study are primarily based on bioinformatics analyses, and the specific biological functions of *MANBA* and *TMTC2*, as well as their regulatory mechanisms in KD, require further validation through *in vitro* cellular experiments and *in vivo* animal models.

## Data Availability

The datasets presented in this study can be found in online repositories. The names of the repository/repositories and accession number(s) can be found in the article/**Supplementary Material**.

## Supplementary material

Supplementary Figure 1 Construction and evaluation of a nomogram prediction model based on MANBA and TMTC2. **(A)** A nomogram for predicting the risk of KD formation. Points are assigned for the expression level of each biomarker (*MANBA* and *TMTC2*), which are summed to obtain a total score corresponding to a probability of disease risk. **(B)** Calibration curve of the nomogram. The x-axis represents the predicted probability of KD development, and the y-axis represents the actual observed frequency. The solid line indicates the performance of the model, and the dashed line represents the ideal reference line. The p-value (0.876) indicates no significant deviation between prediction and observation. **(C)** Decision Curve Analysis (DCA) for the nomogram. The y-axis represents the net benefit. The curves show that using the model for prediction provides a higher net benefit across a range of threshold probabilities compared to the “treat all” or “treat none” strategies.

Supplementary Figure 2 Heatmap showing the correlations between *MANBA* and *TMTC2* and genes associated.
