# Supplementary figures and images for: Identification of potential biomarkers related to mannose metabolism in keloids: analysis of integrated bulk RNA-seq and scRNA-seq
Source: Front Immunol. 2026 Jun 10;17:1711588. doi: 10.3389/fimmu.2026.1711588 (PMC13290920; doi:10.3389/fimmu.2026.1711588)

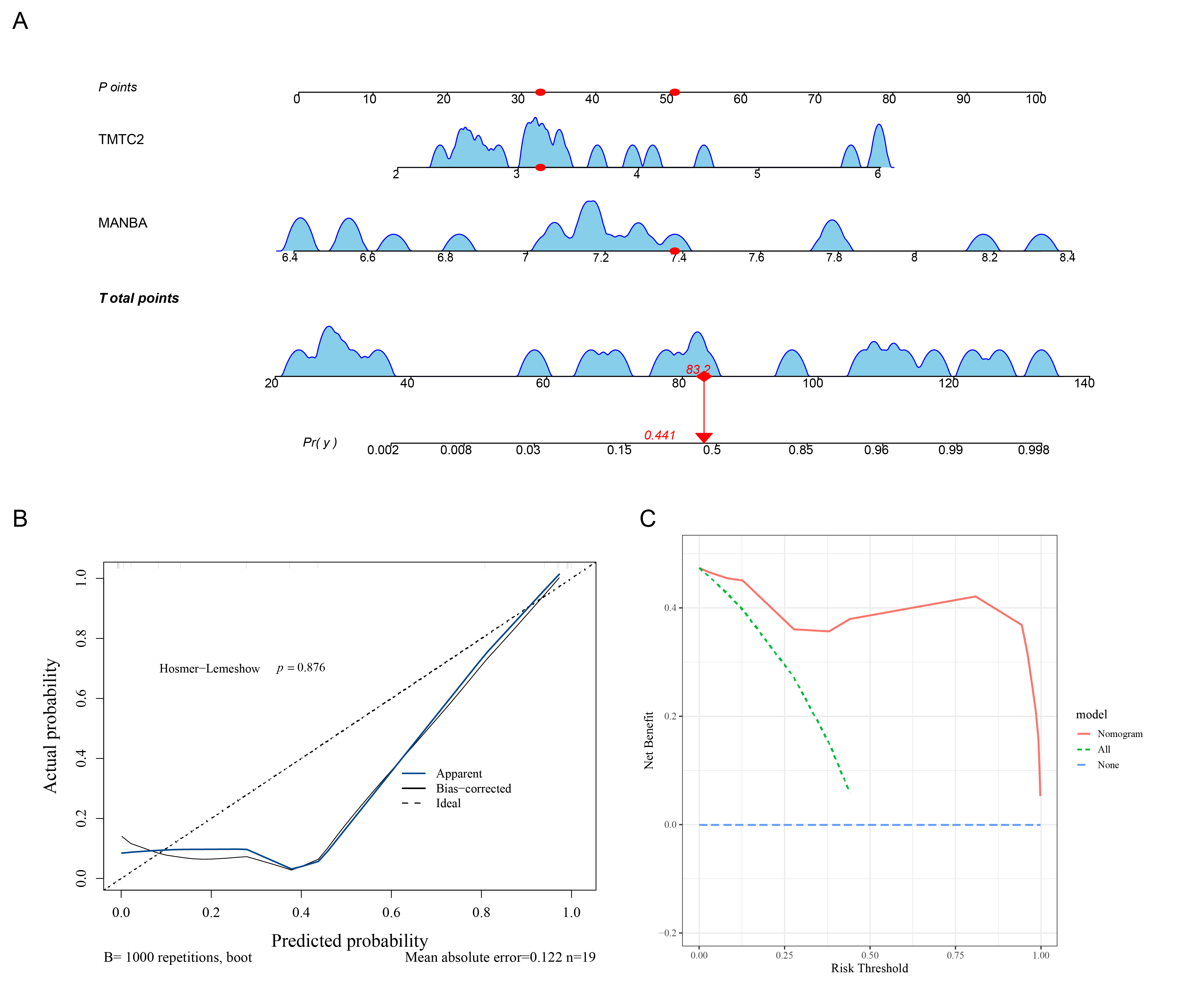

Supplement: Supplementary Figure 1 — Construction and evaluation of a nomogram prediction model based on MANBA and TMTC2. (A) A nomogram for predicting the risk of KD formation. Points are assigned for the expression level of each biomarker (MANBA and TMTC2), which are summed to obtain a total score corresponding to a probability of disease risk. (B) Calibration curve of the nomogram. The x-axis represents the predicted probability of KD development, and the y-axis represents the actual observed frequency. The solid line indicates the performance of the model, and the dashed line represents the ideal reference line. The p-value (0.876) indicates no significant deviation between prediction and observation. (C) Decision Curve Analysis (DCA) for the nomogram. The y-axis represents the net benefit. The curves show that using the model for prediction provides a higher net benefit across a range of threshold probabilities compared to the “treat all” or “treat none” strategies. [file Image1.tif]

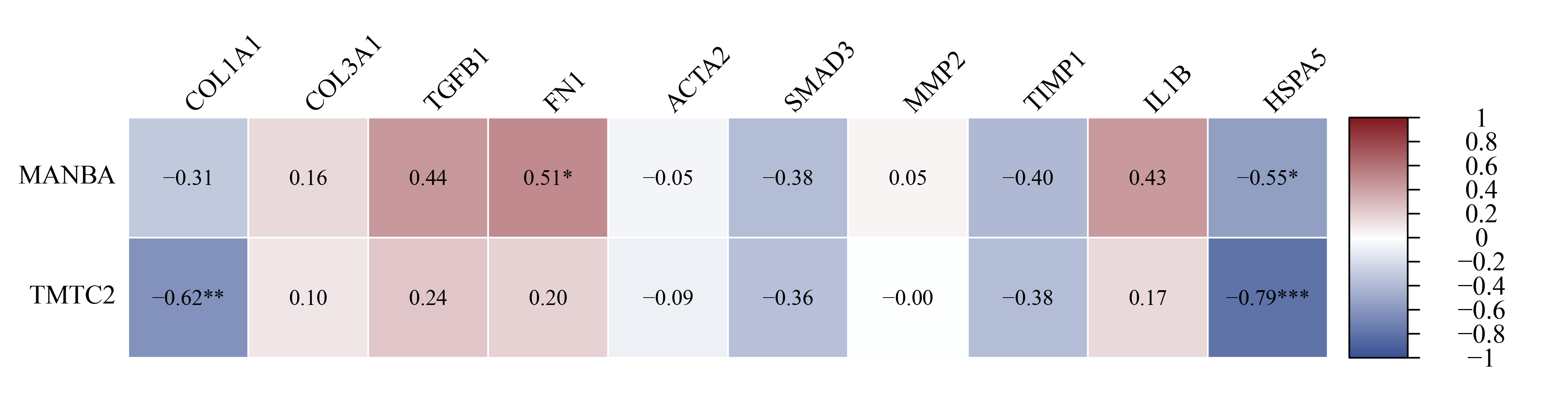

Supplement: Supplementary Figure 2 — Heatmap showing the correlations between MANBA and TMTC2 and genes associated. [file Image2.tif]
